# Retaining young people in a longitudinal sexual health survey: a trial of strategies to maintain participation

**DOI:** 10.1186/1471-2288-10-9

**Published:** 2010-01-28

**Authors:** Marion Henderson, Daniel Wight, Catherine Nixon, Graham Hart

**Affiliations:** 1 MRC Social and Public Health Sciences Unit, Glasgow, UK; 2 Centre for Sexual Health & HIV Research, University College London, London, UK

## Abstract

**Background:**

There is an increasing trend towards lower participation in questionnaire surveys. This reduces representativeness, increases costs and introduces particular challenges to longitudinal surveys, as researchers have to use complex statistical techniques which attempt to address attrition. This paper describes a trial of incentives to retain longitudinal survey cohorts from ages 16 to 20, to question them on the sensitive topic of sexual health.

**Methods:**

A longitudinal survey was conducted with 8,430 eligible pupils from two sequential year groups from 25 Scottish schools. Wave 1 (14 years) and Wave 2 (16 years) were conducted largely within schools. For Wave 3 (18 years), when everyone had left school, the sample was split into 4 groups that were balanced across predictors of survey participation: 1) no incentive; 2) chance of winning one of twenty-five vouchers worth £20; 3) chance of winning one £500 voucher; 4) a definite reward of a £10 voucher sent on receipt of their completed questionnaire. Outcomes were participation at Wave 3 and two years later at Wave 4. Analysis used logistic regression and adjusted for clustering at school level.

**Results:**

The only condition that had a significant and beneficial impact for pupils was to offer a definite reward for participation (Group 4). Forty-one percent of Group 4 participated in Wave 3 versus 27% or less for Groups 1 to 3. At Wave 4, 35% of Group 4 took part versus 25% or less for the other groups. Similarly, 22% of Group 4 participated in all four Waves of the longitudinal study, whereas for the other three groups it was 16% or less that participated in full.

**Conclusions:**

The best strategy for retaining all groups of pupils and one that improved retention at both age 18 and age 20 was to offer a definite reward for participation. This is expensive, however, given the many benefits of retaining a longitudinal sample, we recommend inclusion of this as a research cost for cohort and other repeat-contact studies.

## Background

There is an increasing trend towards lower participation in questionnaire surveys [1]. This affects costs, as more people have to be approached in order to meet the target sample size, and generally reduces representativeness, since participation is biased to particular groups. The challenges are multiplied for longitudinal studies as they aim to retain the same individuals across a number of waves and, after the first wave, individuals who do not respond cannot simply be replaced by substitutes with the same characteristics. Statistical techniques offer strategies which attempt to reduce bias introduced by attrition, for instance weighting and multiple imputation [2]. These options each have strengths and weaknesses and require sophisticated statistical skills to implement [2]. Naturally, whatever statistical approach taken to reduce biases created by sample attrition, external and internal validity is enhanced by retaining as many of the original participants as possible [3].

The authors' interest in response and retention rates was motivated by their goal to maximise these within the *SHARE *cluster randomised trial, which evaluated the effectiveness of teacher-delivered sex education [4]. The trial surveyed young people at average age 14 in school, age 16 in school (except for those who had left school at the earliest legal age allowed (age 16) and who were sent a postal questionnaire to their home), ages 18 and 20 via postal questionnaires. The age range of the different sweeps of the survey covered the transition from school pupils to adults, a transition often associated with geographical mobility which increases the likelihood of attrition within the sample. A further challenge for this study was the sensitive topic of the research, namely sexual health, which could also adversely affect response rates. In order to address these challenges, existing literature in the area of response rates and retention was sourced in order to learn from the work of other researchers.

Past research has shown that there are a number of underlying demographic factors associated with increased likelihood of participating in scientific research and these include being female [3,5,6], of higher socioeconomic status [3,7-9], higher educational attainment, being employed [6], and being married [9]. A number of attempts have been made by researchers to increase participation rates and these include, but are not limited to: reducing the length of questionnaires [10]; using opt-out rather than opt-in consent [11]; using colour and personalising questionnaires and survey correspondence [12,13]; providing a letter of introduction with postal surveys [14]; using telephone or postcard based prompts in postal surveys [15]; and using financial incentives [14,16-20]. This paper will focus upon the effect that offering different types of financial incentives has upon reducing attrition in longitudinal cohorts.

Researchers have explored a range of incentive strategies in order to maximise response rates and the retention of survey participants. Largely, these explorations have been limited to surveys conducted with populations of physicians or other professionals, leaving it unclear whether or not these findings can be generalised to an adolescent population. In a Cochrane review [21] of 292 RCT trials with approximately 260,000 participants, a meta-analysis found that when monetary incentives were used the odds of response doubled, regardless of whether or not the incentive was contingent upon the return of the questionnaire. In a subsequent systematic review [22] it was found that for incentives less than $0.50 each additional $0.01 increased the odds of response by 1%. However, for incentives over $0.50 each additional $0.01 provided a diminishing marginal increase in response. Both of these reviews examined studies that were conducted across both professional and lay persons and no exclusion criteria were applied in reference to the topic of the research. As a result of this, the findings of these reviews may not be generalisable to health related research conducted with adolescents.

Where the use of incentives has been evaluated specifically within the field of health related research, there have been mixed results. In England, Roberts et al. (2000) [20] found that the direct payment of £5 cash incentives increased the response rate to a questionnaire about HRT amongst women aged 40-65 years of age (achieved 67% response rate), whilst inclusion in a prize draw of £50 did not (56.1% response rate). Again based in England, Roberts et al. (2004) [23] in an RCT to evaluate the effect of including a lottery incentive on response rate found that the offer of entry into a lottery style draw for £100 of high street vouchers had no effect on the return rates of postal questionnaires amongst respondents aged 18 years and over (lottery and no incentive conditions both resulted in a 62% response rate). This supported the findings of Aadahl and Jørgensen (2003) [16] from Denmark who demonstrated in another RCT exploring the effect of lottery incentives on response rate that the inclusion of a lottery incentive in their questionnaire on physical activity levels in adults increased the response rate over the first few weeks of the study, but made no overall significant difference to the final response rate (lottery 63% response rate and no incentive 60.4% response rate). Johannsson et al. (1997) [24] based in Norway found that inclusion in a lottery significantly improved the response rate to a postal survey on dietary trends in people aged 16-79 years of age, compared with the offer of no incentive (72% versus 63%). Kalantar and Talley (1999) [18] from Australia found that respondents who received an instant win lottery ticket with a maximum prize of $25,000 (AUS) had a significantly higher response rate than those who did not (75% versus 68%).

Few of the studies described above address the effect of incentives on the response rates to questionnaires amongst adolescent/young adult populations. Of the three studies that we identified, the following findings were observed. Martinson et al. (2000) [19] from USA found that both monetary and lottery style incentives increased the response rate to postal questionnaires about smoking amongst respondents aged between 14 and 17 years of age, with the greatest response rates seen for definite monetary awards (74% response for £15 cash, 69% for token, 63% for prize incentive and 55% with no incentive). The use of incentives did not alleviate the existing gender and age biases in participation, with more girls and younger respondents returning questionnaires. In contrast to the finding that incentives do not increase response rate amongst predicted groups of non-responders, USA based research by Datta et al. (2001) [17] found in an analysis of incentive use in the National Longitudinal Survey of Youth, 1997 Cohort (NLSY97) that the use of monetary incentives can increase the response rates of harder to reach young people, with the size of the incentive being important (the difficult group showing a 76% response to $10 dollars and 78% for $20 - there was not a no incentive condition). Finally, Collins et al. (2000) also based in USA found that for young adults the size of the monetary incentive was more important than whether the incentive is pre-paid or contingent upon the return of a questionnaire, with a 25% increase in payment resulting in a 7% increase in response rate (the highest response rate was 66%).

This paper aims to extend the current literature in three key ways: first, to increase the limited knowledge base about effect of incentives on 18 to 20 year olds in the UK; second, to address the effect of the incentives when collecting highly sensitive data, in this case data relating to sexual attitudes and behaviour, drug use, sexual abuse and domestic violence; and third, the effect of incentives longitudinally. Furthermore, we were also able to test method of completion, offering young people a choice of traditional postal questionnaire, a web-based questionnaire, or a telephone interview.

## Methods

The data for this trial of incentives were collected within the context of a cluster randomized trial to evaluate the effectiveness of teacher-delivered sex education, the *SHARE *study [4]. Ethical permission for the intervention and questionnaire work with pupils was granted by Glasgow University's Ethical Committee for Non-Clinical Research Involving Human Subjects. Following ethical approval, a randomised control trial (RCT) of school sex education was conducted in non-denominational state schools within 15 miles of the main cities in Tayside and Lothian regions of Scotland. Out of 47 schools, 25 agreed to participate.

**Figure 1:**
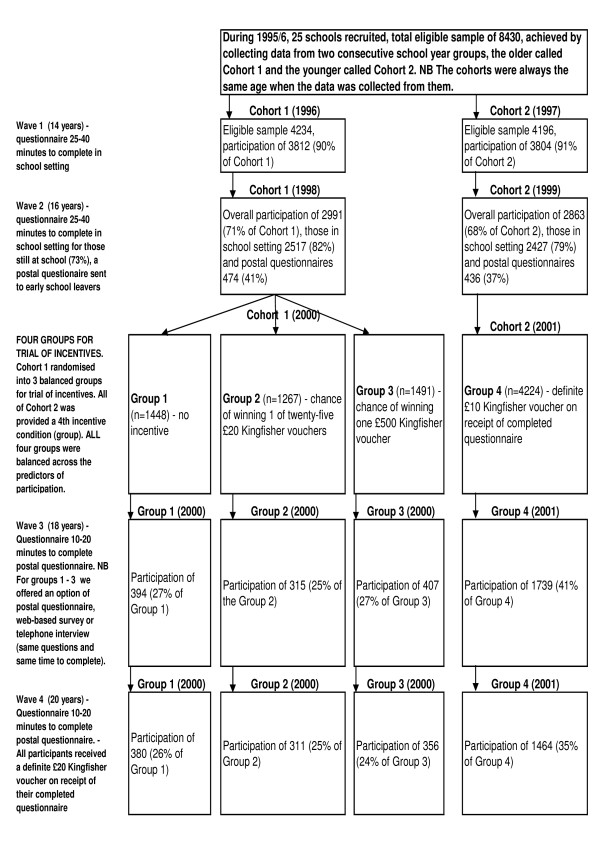
**flow diagram that complements and clarifies the methods and results of this study**.

Figure 1 is a flow diagram for this study and aims to complement and clarify the methods and results of this study. During 1996 and 1997 two successive cohorts of 13 - 14 year olds participated in a baseline survey (mean age 14 years and two months). The 7,616 pupils who participated (of the 8,430 eligible) were representative of 14 year olds throughout Scotland, in terms of parents' social class and the proportion of one-parent households, using 1991 Census data [25]. Data were collected annually from alternate cohorts, such that every two years each cohort of young people was sampled until they were 20 years old in 2002 and 2003 respectively. Wave 1 and Wave 2 were conducted through self-complete questionnaires in schools, although in Wave 2 (mean age 16 years, one month) the 27% who had already left school at the minimum legal age of 16 were sent postal questionnaires. During Wave 3, Cohort 1 was invited to complete a postal questionnaire, a web-based questionnaire, or to do a telephone interview, whilst Wave 3, Cohort 2 and all of Wave 4 was conducted entirely through postal questionnaires. For pupils still at school, pupils' parents could choose to withdraw their child from the study and pupils themselves could opt out of the study. For pupils receiving a postal questionnaire, they were informed that they could withdraw by returning a blank questionnaire.

The issue of attrition to postal questionnaires became clear during the second wave of data collection, when the early school leavers provided a poor response rate to questionnaires (see Results). Given the importance of maximising participation at age 18 and 20, after all the pupils had left school and postal questionnaires were the sole means of data collection, we ran a sub trial to empirically explore the impact of different incentives on participation. Participants at age 18 belonging to Cohort 1 were split into three randomly assigned groups clustered by school. Group 1 received no incentive, Group 2 had a chance of winning one of twenty-five £20 Kingfisher vouchers (odds of approx. 1:300 and with a utility value of 7 pence). Kingfisher vouchers can be spent in a range of stores that sell products such as CDs, DVDs, cosmetics, toiletries and DIY products, but do not sell cigarettes or alcohol. Group 3 had a chance of winning one £500 Kingfisher voucher (odds of approx. 1:1,333 and with a utility value of 38 pence). The following year extra funding was secured to explore the impact (on Cohort 2/Group 4, Wave 4) of offering a definite reward for participation, each pupil was sent a £10 Kingfisher voucher (a utility value of £10) on receipt of their completed questionnaire. Finally, at Wave 4 (the final wave) when participants were aged 20, all participants were offered a definite £15 Kingfisher voucher on receipt of their completed questionnaire (a utility value of £15). In addition, Cohort 1, were given the choice of completing a web-based questionnaire, a telephone interview or a postal questionnaire. At every stage the methods used in this study were balanced across the original arms of the trial (*SHARE *intervention versus control).

### Statistical Methods

There were four stages to this analysis conducted within SPSS version 14 (for descriptive statistics) and MLwiN version 2.14 (for all significance testing, which allowed for clustering at school level as school was the unit of randomisation). First, descriptive statistics were used to describe participation rates for each cohort over the four waves of the trial. Second, the most powerful predictors of non-response were identified. The predictors of non-response were primarily collected at baseline when the participants were 14 years old and the sample had over a 90% participation of the original eligible sample. Third, we tested whether the four conditions (no incentive; chance of winning one of 25 Kingfisher vouchers; chance of winning one £500 Kingfisher voucher; offer of £10 Kingfisher voucher contingent on return of a completed questionnaire) of the trial were balanced across the most powerful predictors of non-response. Fourth, and finally, we formally tested the impact of the four incentive conditions.

## Results

Table 1 complemented by Figure 1 shows the response rates of each cohort at each wave of the survey. When all of the pupils were still at school (Wave 1) over 90% of both cohorts responded. The figure drops for Wave 2 when 27% of the pupils had left school. Wave 2 data were collected from 5,458 young people giving an overall participation rate of 70.4%. There were major differences in the participation rate for those still at school (81%) and early school leavers (39%). At Wave 3 Cohort 1 was randomly assigned to three groups: Group 1 were not offered an incentive; Group 2 had a chance of winning one of twenty-five £20 Kingfisher vouchers (odds of approx. 1:300); and Group 3 had a chance of winning one £500 Kingfisher voucher (odds of approx. 1:1,333) this succeeded in retaining 32.5% of pupils approached (only 26.4% of the original eligible sample). While, at Wave 3 all young people in Cohort 2/Group 4 were offered a £10 Kingfisher voucher on receipt of a completed questionnaires, 48.8% of pupils approached participated and 41.1% of the original eligible sample was retained. In Wave 4 both cohorts (all participants) were offered a £15 Kingfisher voucher on receipt of a completed questionnaire and Groups 1 to 3 retained 37.4% of those approached (24.7% of the original eligible sample), while Group 4 retained 48.7% of those approached (34.4% of the original eligible sample).

**Table 1:** Information relevant to sample retention and response rates for each cohort

Year and cohort (C)	Wave	Method	Age	Eligible start of Wave	With-drawn	Wrong address*	Received quest.	Got data	(%) return rate of those still in study	(%) return rate of original eligible sample
**1996 C1**	**1**	Classroom	13-14	4234	171	N/A^1^	4063	3812	**93.8**	**90.0**
**1997 C2**	**1**	Classroom	13-14	4196	165	N/A^1^	4031	3804	**94.4**	**90.7**
**1998 C1**	**2**	Classroom + postal^2^	15-16	4197	174*	147*	3876	2991	**77.2**	**71.3**
**1999 C2**	**2**	Classroom + postal^2^	15-16	4187	137*	176*	3874	2863	**73.9**	**68.4**
**2000 C1**	**3**	Postal	17-18	3876	127*	313*	3436	1116	**32.5**	**26.4**
**2001 C2**	**3**	Postal	17-18	3874	32*	282*	3560	1739	**48.8**	**41.4**
**2002 C1**	**4**	Mix^3^	19-20	3436	604*	32*	2800	1047	**37.4**	**30.5**
										
**2003 C2**	**4**	Postal	19-20	3560	598*	10*	2952	1439	**48.7**	**40.4**

* When receiving a postal questionnaire, participants were told they could withdraw from the study by returning a blank questionnaire in the pre-paid envelope. Wrongly addressed questionnaires were returned by the postal service in their original envelopes. However, it is possible that new residents opened the envelope and then returned the blank questionnaire in the pre-paid envelope. Thus, there is a possibility of some of the 'withdrawn' numbers actually being due to a 'wrong address'.

^1 ^The reason for N/A in this cell is that there was no possibility of a 'wrong address,' as all the questionnaires were completed in school classrooms, no pupils had left school and thus no pupils required a questionnaire to be posted out to them.

^2^In 1998, overall participation of 2991 (71% of Cohort 1), those in school setting 2517 (82%) and postal questionnaires 474 (41%). In 1999, overall participation of 2863 (68% of Cohort 2), those in school setting 2427 (79%) and postal questionnaires 436 (37%).

^3^The 'mix' was a choice of completing a web-based questionnaire, a telephone interview or a postal questionnaire.

Table 2 illustrates that only a very small proportion of pupils from the original eligible sample failed to complete a questionnaire in any wave (3%). Over half of pupils participated in two or three waves of the survey. It is of note that Group 4, which from age 18 (Wave 3) were offered a definite reward for participation had 22% of pupils participating in all 4 waves of the survey, whereas the other three groups, that did not receive a definite reward until age 20, had only 14.1% of pupils participating in all 4 waves.

**Table 2:** Distributions for participating in different numbers of waves of the survey

	Group				
	Group 1	Group 2	Group 3	Group 4	Total
No. of waves in which pupils participated	no incentive	chance of winning 1 of twenty-five £20 Kingfisher vouchers	chance of winning one £500 Kingfisher voucher	definite £10 Kingfisher voucher on receipt of completed questionnaire	
	n (%)	n (%)	n (%)	n (%)	
**Zero participation (eligible pupils who never participated in any wave)**	47 (3.2)	34 (2.7)	48 (3.2)	143 (3.4)	272
					
**Participated in 1 wave only of the study**	340 (23.5)	318 (25.1)	405 (27.2)	958 (22.7)	2021
					
**Participated in 2 waves of the study**	589 (40.7)	533 (42.1)	612 (41.0)	1399 (33.1)	3133
					
**Participated in 3 waves of the study**	245 (16.9)	200 (15.8)	241 (16.2)	781 (18.5)	1467
					
**Full participation in ALL 4 waves of the study**	227 (15.7)	182 (14.4)	185 (12.4)	943 (22.3)	1537
					
**Total**	1448	1267	1491	4224	8430

While the descriptive statistics described above suggest that a definite reward for participation is helpful, before formally testing this it is necessary to assess whether the four incentive conditions were balanced for key predictors of non-response. The predictors of non-response had been explored within *SHARE *when developing inverse probability weights for use when analysing data from age 16, Wave 2. The weighting strategy has been described and used within a number of papers arising from the *SHARE *study [26,27]. Table 3 below shows the impact on response rate of the most powerful predictors of non-response at 16, 18 and 20 years of age in the *SHARE *study. These predictors of non-response were: being male; at age 14 father was a manual worker (blue-collar worker); mother was a manual worker; not living with both parents; low parental monitoring; more than £20 per week to spend; was drunk once a month or more frequently; and finally, measured at age 16, leaving school early (at the minimum legal age). It should be noted that receiving the *SHARE *teacher delivered sex education was not related to questionnaire participation.

**Table 3:** Univariate effects of different predictors of participation upon respondent retention at ages 16, 18 and 20 (as expected from the literature, all the predictors were significant, except for receiving the *SHARE *teacher-delivered sex education at school)

		Completed questionnaire at age 16 (Wave 2)		Completed questionnaire at age 18 (Wave 3)		Completed questionnaire at age 20 (Wave 4)	
		Odds ratio	(95% CI)	Odds ratio	(95% CI)	Odds ratio	(95% CI)
**All pupils (N = 8430)**							
**Sex**	female	1.94	(1.76, 2.13)	2.65	(2.42, 2.91)	2.37	(2.16, 2.61)
	male	1		1		1	
							
**Father manual versus non manual worker**	non-manual	2.27	(2.01, 2.58)	1.49	(1.34, 1.66)	1.32	(1.18, 1.48)
	missing data	0.60	(0.54, 0.67)	0.82	(0.73, 0.92)	0.78	(0.70, 0.88)
	manual	1		1		1	
							
**Mother manual versus non manual worker**	non-manual	1.99	(1.76, 2.26)	1.34	(1.19, 1.51)	1.26	(1.11, 1.42)
	missing data	0.69	(0.61, 0.77)	0.79	(0.70, 0.89)	0.78	(0.69, 0.89)
	manual	1		1		1	
							
**Family composition**	lives with mum only	0.53	(0.47, 0.59)	0.80	(0.72, 0.89)	0.74	(0.66, 0.82)
	lives with dad only	0.40	(0.32, 0.51)	0.48	(0.36, 0.63)	0.45	(0.34, 0.61)
	lives with neither parent	0.31	(0.21, 0.46)	0.41	(0.25, 0.67)	0.55	(0.34, 0.89)
	missing data	0.03	(0.02, 0.05)	0.23	(0.16, 0.31)	0.26	(0.19, 0.37)
	lives with both parents	1		1		1	
							
**Parental monitoring**	low parental monitoring	0.69	(0.63, 0.76)	0.63	(0.57, 0.69)	0.69	(0.63, 0.76)
	high parental monitoring	1		1		1	
							
**Pocket Money**	more than £20 per week			0.87	(0.8, 0.89)	0.89	(0.87, 0.90)
	less than £20 per week	N/A*		1		1	
							
Sexual intercourse by 16 years*	has had sexual intercourse			0.91	(0.81, 1.01)	0.87	(0.78, 0.98)
	missing data			0.26	(0.23, 0.29)	0.30	(0.27, 0.34)
	has not had sexual intercourse	N/A*		1		1	
							
**Early school leaving status**	leavers	0.15	(0.14, 0.17)	0.55	(0.50, 0.62)	0.65	(0.58, 0.73)
	non-leavers	1		1		1	
							
**Drunkenness**	gets drunk once a month or more	N/A*		0.74	(0.67, 0.81)	0.81	(0.73, 0.90)
	never or rarely gets drunk			1		1	
							
**The *SHARE *teacher delivered sex education intervention**	Received *SHARE*	0.91	(0.82, 1.01)	0.92	(0.84, 1.01)	0.92	(0.84, 1.01)
	Not received *SHARE*	1		1		1	

*Some analysis is N/A at age 16. This is because the variables with N/A were themselves collected at age 16, thus we can not look at the impact of these on participation rates at 16, because we do not know the information required for the non-responders. The other variables we were able to use were collected at age 14, before the individuals did not respond, or in the case of early school leaving were provided to us by the school the participants' attended.

The next step was to test whether randomisation had helped to generate 4 groups that were matched across the predictors of non participation. Table 4 shows that the 4 groups were balanced (no statistical difference) for all of the predictors of participation, namely, gender, occupational classification of father, occupational classification of mother, family composition, parental monitoring, spending money, early school leavers and frequency of drunkenness. This means that the randomisation of the Groups 1 to 3 was successful and also balanced with Group 4/Cohort 2. The balance with Group 4/Cohort 2 was expected given that the Cohorts are simply two consecutive year groups of pupils from the same schools, geographical areas and had all their data collected at the same time of year and at the same age.

**Table 4:** Balance across the significant predictors of participation for the four incentive conditions (Groups 1 to 4)

	Group 1	Group 2	Group 3	Group 4	
	no reward n(%)	25 prizes of £20 n(%)	one £500 prize n(%)	£10 reward on receipt of completed questionnaire n(%)	Total N
**Total N**	1448	1267	1491	4224	8430
					
**Gender (NS^1^)**					
male	687 (47.4)	638 (50.4)	687 (46.1)	2033 (48.1)	4045
female	713 (49.2)	590 (46.6)	747 (50.1)	2019 (47.8)	4069
missing data	48 (3.3)	39 (3.1)	57 (3.8)	172 (4.1)	316
					
**Father manual versus non manual worker (NS^1^)**					
non-manual	517 (35.7)	417 (32.9)	511 (34.3)	1411 (33.4)	2856
manual	493 (34.0)	441 (34.8)	510 (34.2)	1410 (33.4)	2854
missing data	438 (30.2)	409 (32.3)	470 (31.5)	1403 (33.2)	2720
					
**Mother manual versus non manual worker (NS^1^)**					
non-manual	604 (41.7)	511 (40.3)	618 (41.4)	1777 (42.1)	3510
manual	343 (23.7)	277 (21.9)	324 (21.7)	953 (22.6)	1897
missing data	501 (34.6)	479 (37.8)	549 (36.8)	1494 (35.4)	3023
					
**Family composition (NS^1^)**					
lives with both parents	960 (66.3)	829 (65.4)	1016 (68.1)	2791 (66.1)	5596
lives with mum only	375 (25.9)	328 (25.9)	329 (22.1)	1027 (24.3)	2059
lives with dad only	50 (3.5)	54 (4.3)	54 (3.6)	149 (3.5)	307
lives with neither parent	12 (0.8)	12 (0.9)	22 (1.5)	57 (1.3)	103
missing data	51 (3.5)	44 (3.5)	70 (4.7)	200 (4.7)	365
					
**Parental monitoring (NS^1^)**					
high parental monitoring	652 (46.6)	513 (41.8)	649 (45.3)	1858 (45.9)	3672
low parental monitoring	748 (53.4)	715 (58.2)	785 (54.7)	2194 (54.1)	4442
					
**Pocket Money (NS^1^)**					
less than £20 per week	681 (48.6)	528 (43.0)	687 (47.9)	1790 (44.2)	3686
more than £20 per week	719 (51.4)	700 (57.0)	747 (52.1)	2262 (55.8)	4428
					
**Sexual intercourse by 16 years of age (NS^1^)**					
has had sexual intercourse	373 (36.6)	334 (37.4)	404 (37.4)	1057 (36.9)	2168
has not had sexual intercourse	637 (62.4)	544 (60.9)	666 (61.7)	1767 (61.7)	3614
missing data	8 (0.8)	15 (1.7)	10 (0.9)	39 (1.4)	72
					
**Early school leaving status (NS^1^)**					
non-leavers	1053 (72.7)	874 (69.0)	1135 (76.1)	3054 (72.3)	6116
leavers	395 (27.3)	393 (31.0)	356 (23.9)	1170 (27.7)	2314
					
**Drunkenness (NS^1^)**					
never or rarely gets drunk	867 (59.9)	687 (54.2)	761 (51.0)	2430 (57.5)	4745
gets drunk once a month or more	481 (33.2)	486 (38.4)	466 (31.3)	1309 (31.0)	2742
Missing data	100 (6.9)	94 (7.4)	264 (17.7)	485 (11.5)	943

^1 ^All results were NS at the 95% level of confidence.

Table 5 shows the results of two multivariate logistic regressions that were undertaken to test the effects of the incentives for pupils at ages 18 and 20. Before incentives (baseline) at Wave 2 (age 16) there was no significant difference in participation between any of the four groups. After implementing the different incentives, results show that at both 18 and 20 years, Group 4, where respondents received a £10 voucher on receipt of a completed questionnaire at Wave 3 (age 18), showed a significantly increased likelihood of response.

**Table 5:** Effect of incentives at baseline (age 16), age 18 and age 20 year olds (the results that are statistically significant are in bold)

Covariate	Sub-group	Age 16 (baseline for incentives)		Age 18		Age 20	
				Odds ratio	(95% CI)	Odds ratio	(95% CI)
**All pupils (N = 8430) Type of reward offered at age 18**	25 prizes of £20 (Group 2)	1.01	(0.86, 1.19)	0.89	(0.74, 1.05)	0.91	(0.77, 1.09)
							
	One £500 prize (Group 3)	1.11	(0.95, 1.30)	1.00	(0.85, 1.18)	0.88	(0.75, 1.04)
							
	£10 voucher on receipt of completed questionnaire (Group 4)	.89	(0.78, 1.02)	**1.87**	**(1.64, 2.13)**	**1.49**	**(1.30, 1.70)**
	No reward (Group 1)	1		1		1	

Finally, Table 6 shows the uptake of our offer to complete the questionnaire by postal questionnaire (pen and paper), web or by telephone (free of charge). This choice was offered to Groups 1 to 3 participants at Wave 4 (age 20), to see if offering alternative modes of completing the questionnaire would improve the participation rate over that achieved in Wave 3. It is clear that the overwhelming proportion of pupils opted for a questionnaire to be posted to them.

**Table 6:** Wave 4 Cohort 1 participants' uptake of offer to complete the questionnaire by pen and paper, web or telephone interview

Questionnaire mode	N (%)
Postal (pen and paper)	759 (72.5)
Web based	263 (25.1)
Telephone administered	20 (1.9)
Total complete questionnaires	1047

## Discussion

The results confirm the challenge of retaining a longitudinal sample by postal questionnaire, especially when young people are making the transition from secondary school to their adult life and are geographically mobile. By the end of Wave 4 (age 20) we had retained a quarter of Groups 1 to 3 and a third of Group 4. The evidence shows that the difference in retention rate was associated with the incentive conditions we evaluated in the analysis for this paper. Group 1 was offered no incentive, Group 2 a chance of winning one of twenty-five £20 Kingfisher vouchers and Group 3 a chance of winning one £500 Kingfisher voucher. When formally tested none of these three strategies were successful at increasing response rates. This finding is in line with other (not youth specific) evaluations of lottery incentives [16,20,24]. It was clear that the best strategy for retaining all groups of pupils and one that improved retention at both age 18 and age 20 was to offer a definite reward for participation. This finding is in line with that of Martinson et al. (2000) who found that offering a definite monetary award for completion of a smoking questionnaire by 14 to 17 year olds yielded the largest increase in response rate [19]. Our age 16 (Wave 2) participation rate (70%) was comparable with Martinson et al.'s [19] highest response rate of 74% for 14-17 year olds.

No studies were identified that collected such sensitive, sexual health, data and that covered four Waves at the same ages as the *SHARE *RCT. The findings of this study therefore provide unique evidence on retaining young people in sensitive research over a transition period of their lives.

If the strategy of offering a definite reward for participation were to be implemented earlier at age 15/16, there may be a tension in offering a reward to early school leavers while the others are still being surveyed at school, as those still at school may feel their previous school-mates are being offered something simply because they left early, while they are being disadvantaged for staying on at school. However, the benefits of retaining leavers at an early stage may outweigh that tension. Our participation rates for pupils still in a school setting were very high (Wave 1 and vast majority of Wave 2 participants), which suggests there would be no added benefit of paying school based pupils for completing questionnaires. In addition, school students frequently complete questionnaires and sit tests without any cash incentive. For those that have left the school setting, a voucher/cash incentive could be viewed as paying participants for their time that they could otherwise be using to do paid work.

A limitation of our study was that we randomised Groups 1 to 3 who all belonged to Cohort 1, while exactly a year later all of Cohort 2 became Group 4. Ideally, we would have randomised all four groups. The reason for not randomising all four groups was due to inadequate funding to allow us to test for a definite reward for participation when we randomised Cohort 1. We succeeded in securing the additional funding the following year. However, the two cohorts are simply two consecutive year groups of pupils from the same schools, which were randomly assigned at school level within the context of the *SHARE *RCT [4], data were always collected at the same time of year (the Autumn/Fall term), the participants were the same age when completing questionnaires and no significant effect of Cohort has ever been detected within the *SHARE *RCT [4]. Thus, there is no reason to expect Group 4/Cohort 2 not to be balanced across the predictors of participation with Groups 1 to 3. The analysis shown in Table 4 confirms that all four groups were balanced across all the predictors of participation. Thus, while this is not a conventional randomised trial, it is a fair trial of the four different incentives explored in this paper.

The uptake of our offer to complete the questionnaire by web or by telephone (free of charge) was very low and did not seem worth the substantial costs of setting-up these options. In 2002 and 2003 the overwhelming preference was to complete by paper and pen. However, since access to broadband continues apace, it might be worth exploring the web option again in the future.

## Conclusions

The best strategy for retaining all groups of pupils beyond school and one that improved retention at both age 17/18 and age 19/20 was to offer a definite reward for participation. While this is expensive, given the many benefits of retaining a longitudinal sample, we recommend inclusion of this as a research cost for cohort and other repeat-contact studies.

## Competing interests

The authors declare that they have no competing interests.

## Authors' contributions

MH, DW and GH designed the trial of incentives, while MH and DW collected the data. MH analyzed the data. DW and CN commented on the analysis. MH and CN drafted the paper and MH revised subsequent drafts based on co-authors' comments. MH, DW, CN and GH commented on subsequent drafts of the paper and all authors have read and approved the final manuscript.

## Pre-publication history

The pre-publication history for this paper can be accessed here:

http://www.biomedcentral.com/1471-2288/10/9/prepub

## References

[B1] Galea S Tracy M Participation Rates in Epidemiologic Studies Annals of Epidemiology 2007 17 9 643 653 10.1016/j.annepidem.2007.03.013 17553702

[B2] Carpenter J Kenward M Missing Data http://www.lshtm.ac.uk/msu/missingdata/

[B3] Collins RL Ellickson PK Hays RD McCaffrey DF Effects of Incentive Size and Timing on Response Rates to a Follow-up Wave of a Longitudinal Mailed Survey Evaluation Review 2000 24 4 347 363 10.1177/0193841X0002400401 11009863

[B4] Wight D Raab GM Henderson M Abraham C Buston K Hart G Scott S Limits of teacher delivered sex education: interim behavioural outcomes from randomised trial BMJ 2002 324 7351 1430 10.1136/bmj.324.7351.1430 12065268 PMC115856

[B5] Dunn KM Jordan K Lacey RJ Shapley M Jinks C Patterns of Consent in Epidemiologic Research: Evidence from Over 25,000 Responders Am J Epidemiol 2004 159 11 1087 1094 10.1093/aje/kwh141 15155293

[B6] Eagan TML Eide GE Gulsvik A Bakke PS Nonresponse in a community cohort study: Predictors and consequences for exposure-disease associations Journal of Clinical Epidemiology 2002 55 775 781 10.1016/S0895-4356(02)00431-6 12384191

[B7] Cunradi CB Moore R Killoran M Ames G Survey Nonresponse Bias Among Young Adults: The Role of Alcohol, Tobacco, and Drugs Substance Use & Misuse 2005 40 2 171 185 10.1081/ja-200048447 15770883

[B8] Hille ETM Elbertse L Gravenhorst JB Brand R Verloove-Vanhorick SP on behalf of the Dutch P-CSG Nonresponse Bias in a Follow-up Study of 19-Year-Old Adolescents Born as Preterm Infants Pediatrics 2005 116 5 e662 666 10.1542/peds.2005-0682 16263980

[B9] Partin MR Malone M Winnett M Slater J Bar-Cohen A Caplan L The impact of survey nonresponse bias on conclusions drawn from a mammography intervention trial Journal of Clinical Epidemiology 2003 56 867 873 10.1016/S0895-4356(03)00061-1 14505772

[B10] Dillman DA Sinclair MD Clark JR Effects of questionnaire length, respondent-friendly design, and a difficult question on response rates for occupant-addressed census mail surveys Public Opin Q 1993 57 289 304 10.1086/269376

[B11] Junghans C Feder G Hemingway H Timmis A Jones M Recruiting patients to medical research: double blind randomised trial of "opt-in" versus "opt-out" strategies BMJ 2005 331 7522 940 10.1136/bmj.38583.625613.AE 16157604 PMC1261191

[B12] Etter J-F Cucherat M Perneger TV Questionnaire Color and Response Rates to Mailed Surveys: A Randomizedtrial Anda Meta-Analysis Eval Health Prof 2002 25 2 185 199 10.1177/01678702025002004 12026752

[B13] Moss VDaW BR Do personalization and postage make a difference on response rates to surveys of professional populations Psychol Rep 1991 68 692 694 10.2466/PR0.68.2.692-694

[B14] Senn CY Desmarais S Verberg N Wood E Sampling the Reluctant Participant: A Random-Sample Response-Rate Study of Men and Sexual Coercion Journal of Applied Social Psychology 2006 30 1 96 105 10.1111/j.1559-1816.2000.tb02307.x

[B15] Bruce T Salkeld G Short L Solomon M Ward J A randomised trial of telephone versus postcard prompts to enhance response rate in a phased population-based study about community preferences Australian and New Zealand Journal of Public Health 2000 24 4 456 457 10.1111/j.1467-842X.2000.tb01615.x 11011481

[B16] Aadahl M Jorgensen M The Effect of Conducting a Lottery on Questionnaire Response Rates: A Randomised Controlled Trial European Journal of Epidemiology 2003 18 10 941 944 10.1023/A:1025826921030 14598924

[B17] Datta AR Horrigan MW Walker JR Evaluation of a Monetary Incentive Payment Experiment in the National Longitudinal Survey of Youth, 1997 Cohort Presented at the 2001 Federal Committee on Statistical Methodology Conference

[B18] Kalantar JS Talley NJ The effects of lottery incentive and length of questionnaire on health survey response rates: a randomised study Journal of Clinical Epidemiology 1999 52 11 1117 1122 10.1016/S0895-4356(99)00051-7 10527007

[B19] Martinson BC Lazovitch D Lando HA Perry CL McGovern PG Boyle RG Effectiveness of Monetary Incentives for Recruiting Adolescents to an Intervention Trial to Reduce Smoking Preventative Medicine 2000 31 706 713 10.1006/pmed.2000.0762 11133338

[B20] Roberts PJ Roberts C Sibbald B Torgerson DJ The effect of a direct payment or lottery on questionnaire response rates: a randomised control trial Journal of Epidemiology and Community Health 2000 54 71 72 10.1136/jech.54.1.71 10692967 PMC1731530

[B21] Edwards P Roberts I Clarke M DiGuiseppi C Pratap RW Kwan I Increasing response rates to postal questionnaires: systematic review BMJ 2002 324 1183 1191 10.1136/bmj.324.7347.1183 12016181 PMC111107

[B22] Edwards P Cooper R Roberts I Frost C Meta-analysis of randomised trials of monetary incentives and response to mailed questionnaires Journal of Epidemiology and Community Health 2005 59 11 987 999 10.1136/jech.2005.034397 16234429 PMC1732953

[B23] Roberts LM Wilson S Roalfe A Bridge P A Randomised Control Trial to Determine the Effect on Response of Including a Lottery Incentive in Health Surveys BMC Health Services Research 2004 4 30 15533256 10.1186/1472-6963-4-30 PMC534094

[B24] Johansson L Solvoll K Opdahl SA Bjorneboe G-E Aa Drevon CA Response rates with different distribution methods and reward and reproducibility of a quantitative food frequency questionnaire European Journal of Clinical Nutrition 1997 51 6 343 353 10.1038/sj.ejcn.1600410 9192190

[B25] Henderson M Wight D Raab G Abraham C Buston K Hart G Scott S Heterosexual risk behaviour among young teenagers in Scotland Journal of Adolescence 2002 25 483 494 10.1006/jado.2002.0493 12234555

[B26] Henderson M Ecob R DW Abraham C What explains between-school differences in rates of smoking? BMC Public Health 2008 8 218 10.1186/1471-2458-8-218 18570635 PMC2442834

[B27] Parkes A Henderson M Wight D Do sexual health services encourage teenagers to use condoms? A longitudinal study Journal of Family Planning and Reproductive Health Care 2005 31 4 271 280 16274548 10.1783/jfp.31.2.271

